# Introduction of Exercise Therapy Using Hybrid Assistive Limb (HAL®) Lumbar Type for Diabetes Patients: A Pilot Study

**DOI:** 10.7759/cureus.90140

**Published:** 2025-08-15

**Authors:** Tomokazu Abe, Kousei Miura, Tsuyoshi Saito, Naoki Kayanoma, Yuuma Furukawa, Yoshihiro Yasunaga, Masashi Yamazaki, Masataka Kusunoki

**Affiliations:** 1 Graduate School of Medicine, Nagoya University, Nagoya, JPN; 2 Department of Orthopaedic Surgery, Institute of Medicine, University of Tsukuba, Tsukuba, JPN; 3 Akishima Clinic, Medical Corporation Oda-kai, Nagoya, JPN; 4 Nagoya Diabetes Exercise Therapy Center, Apollon Co. Ltd, Nagoya, JPN; 5 Nagoya Diabetes Exercise Therapy Center, Apollon Co. Ltd., Nagoya, JPN; 6 Research Devision, Cyberdyne Inc., Tsukuba, JPN; 7 Center for Comprehensive Health and Sports Science, Nagoya University, Nagoya, JPN

**Keywords:** diabetes, errorless motor learning effect, exercise therapy, hybrid assistive limb, wearable exoskeleton

## Abstract

Background: Exercise therapy is essential for diabetes management, but adherence remains challenging for many patients. The hybrid assistive limb (HAL) lumbar type is a wearable robot that has shown effectiveness in various conditions, yet its application in diabetes patients has not been investigated.

Objective: To evaluate the feasibility and safety of exercise therapy using the HAL lumbar type for patients with diabetes and to exploratorily investigate its effects on physical function and metabolic parameters.

Methods: This pilot, prospective, single-arm study included nine patients with diabetes (mean age: 69.00±13.23 years). Participants underwent an exercise program using the HAL lumbar type, consisting of eight sessions of 60 minutes each. The program included stretching, squatting, and walking exercises, performed 1-2 times per week, with the goal of completion within 4 to 8 weeks. The primary outcome was the Sit-To-Stand test (STS test). Secondary outcomes included changes in body weight, body mass index (BMI), and metabolic parameters.

Results: All participants completed the intervention without adverse events. Significant improvements were observed in the STS test (15.86±3.87 to 10.43±2.27 seconds, p<0.005), body weight (72.68±15.73 to 70.71±15.15 kg, p<0.05), and lipid metabolism parameters including total cholesterol (200.11±32.85 to 182.67±21.45 mg/dL, p<0.05) and LDL cholesterol (114.00±28.51 to 101.56±24.52 mg/dL, p<0.005).

Conclusion: Exercise therapy using the HAL lumbar type was safely implemented in diabetes patients and showed potential benefits in improving physical function and lipid metabolism. These findings suggest that HAL-assisted exercise therapy might be a promising new approach for diabetes management.

## Introduction

Diabetes is increasingly recognized as a major risk factor for stroke and cardiovascular disease. The global prevalence of diabetes, including those at risk, continues to rise, with an estimated 537 million adults living with diabetes worldwide as of 2021 [1]. This trend emphasizes the urgent need for comprehensive approaches to prevention, early treatment, and prevention of complications.

Diabetes management in middle-aged and older adults is particularly crucial as it carries significant risks of severe complications, including the need for dialysis and lower limb amputation [2-4]. The basic therapeutic approach to diabetes comprises three main components: pharmacological therapy, dietary management, and exercise therapy. Among these, exercise therapy plays a vital role in affecting whole-body metabolic function. However, adherence to exercise regimens often proves challenging due to various factors, including time constraints in daily life and low self-efficacy [5]. While various approaches such as multicomponent exercise and High-Intensity Interval Training (HIIT) have been proposed [6,7], the difficulty in maintaining long-term participation remains a primary obstacle. This highlights the need for novel interventions that can enhance both the efficiency of and adherence to exercise programs. Advanced assistive technologies may offer a potential solution to this challenge.

With the advancement of Internet of Things technology, various devices have been developed to assist exercise therapy [8]. The Hybrid Assistive Limb (HAL®) Lumbar Type, a wearable cyborg system, has demonstrated effectiveness in various conditions, including neuromuscular diseases, musculoskeletal disorders, frailty, and care-dependent individuals [9-11]. However, its application in diabetes patients has not been previously investigated. Therefore, this pilot study was designed with the primary objective to evaluate the feasibility and safety of a short-term, low-intensity exercise program using the HAL® Lumbar Type in patients with diabetes. As a secondary, exploratory objective, we also investigated the effects of this intervention on physical function and metabolic parameters.

## Materials and methods

Study design

This study was designed as a pilot, prospective, single-arm rehabilitation study using the HAL lumbar type for patients with diabetes. The study was conducted between August 2023 and February 2024 and was approved by the Ethics Committee of Oda Medical Corporation (approval number 2023-02). The study was conducted in accordance with the principles of the Declaration of Helsinki.

Participants

Nine patients diagnosed with diabetes by specialized physicians at a diabetes clinic were enrolled in the study. The inclusion criteria were (1) diagnosis of diabetes by a specialist physician and (2) ability to understand and follow the exercise program instructions. Patients with severe cognitive or motor function impairments that would interfere with daily activities were excluded. The participants' characteristics were as follows: mean age 69.00 ± 13.23 years (men: 71.5 ± 4.50 years, women: 67.00 ± 17.98 years), mean height 162.36 ± 9.84 cm (men: 168.80 ± 7.75 cm, women: 157.20 ± 8.58 cm), mean weight 72.68 ± 15.73 kg (men: 84.93 ± 15.71 kg, women: 62.88 ± 6.29 kg), and mean BMI 27.38 ± 4.02 (men: 29.65 ± 4.33, women: 25.56 ± 3.00).

HAL lumbar type description

**Figure 1:**
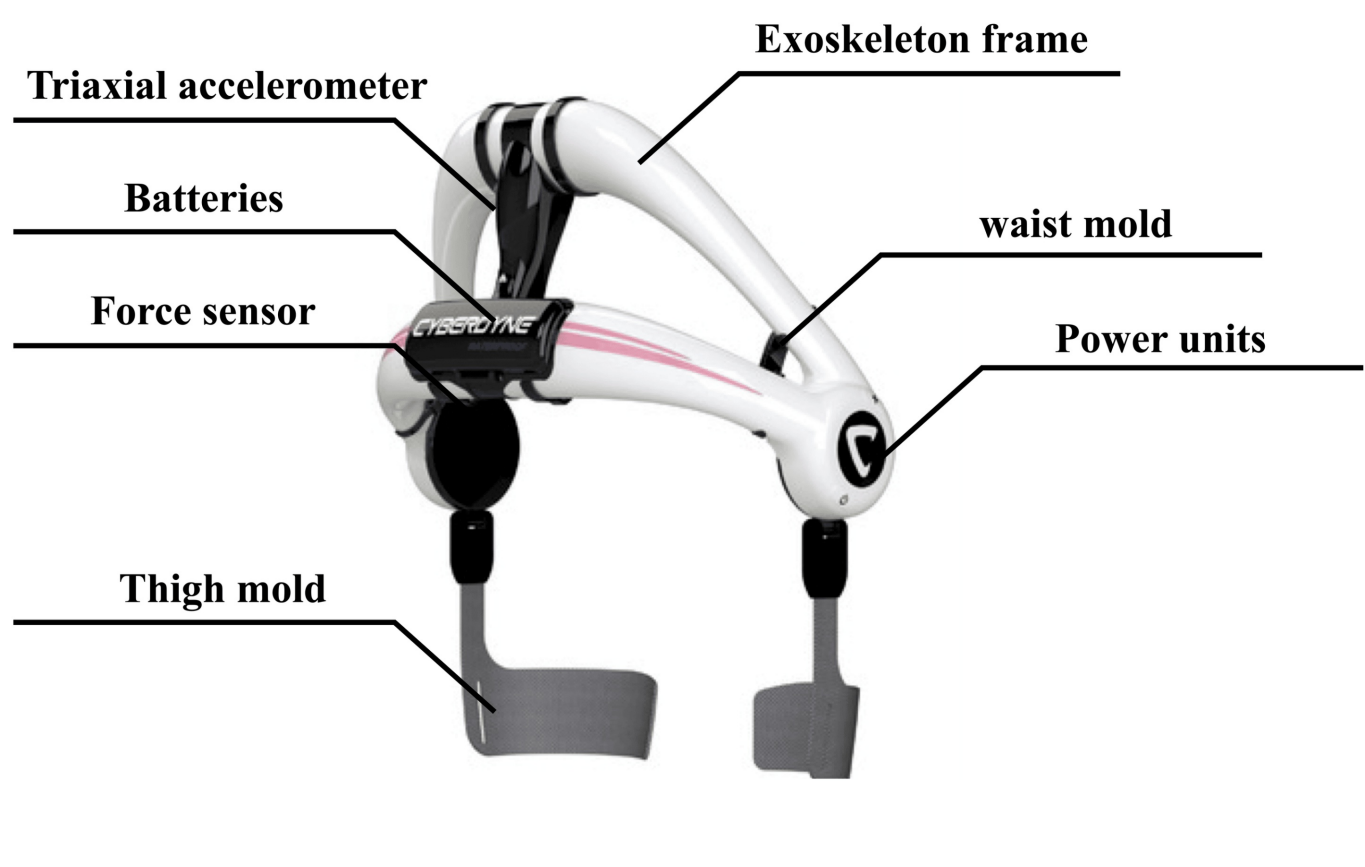
Wearable cyborg HAL® lumber type HAL: Hybrid Assistive Limb The image is obtained from http://www.cyberdyne.jp/.

The HAL lumbar type (CYBERDYNE Inc., Tsukuba, Japan) consists of a lumbar and thigh mold, angle sensors, triaxial accelerometer sensors, and power units integrated into an exoskeleton frame (Figure 1).

**Figure 2:**
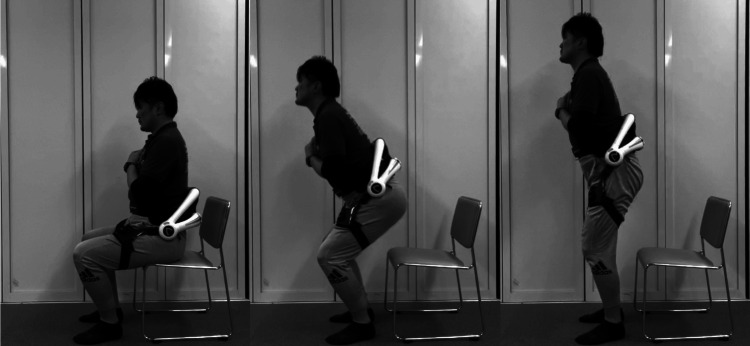
Using the HAL® Lumbar Type (Squatting) HAL: Hybrid Assistive Limb

During squatting exercises, the device reduces the load on the wearer's lower back through belts securing the lumbar and thigh regions, assisting hip extension torque from the trunk flexion phase through the buttocks separation phase to the knee extension phase (Figure 2).

The device’s assistance is controlled by angle sensors, triaxial accelerometer sensors, and bioelectrical signals from the lumbar erector spinae muscles. Two control modes are available: Cybernic Autonomous Control (CAC), which uses only angle and accelerometer sensors, and Cybernic Voluntary Control (CVC), which incorporates bioelectrical signals. The assist torque can be adjusted in five levels. In this study, the CAC mode was used with maximum assist torque.

Intervention protocol

The intervention protocol was standardized according to a pre-defined manual, and all sessions were supervised by a single physical therapist proficient in the operation of HAL.

The program consisted of a total of eight sessions. The frequency was set at 1-2 sessions per week to accommodate patient schedules and minimize their burden. The protocol aimed for completion of all eight sessions within 4 weeks, but allowed for an extension up to 8 weeks based on participants' convenience.

Each session lasted 60 minutes and consisted of: Stretching exercises (30 minutes, serving as a warm-up); Squatting exercises (15 minutes); Walking exercises (15 minutes). A 5-minute cool-down with light stretching was performed after each session.

Exercise intensity was monitored through heart rate measurements, with a target range of 110-120 beats per minute. If a participant's heart rate exceeded 120 bpm, the exercise was temporarily paused until the heart rate returned to the target range before resuming. Heart rate was measured four times during each session (pre-exercise, during squatting, during walking, and post-exercise).

Outcome measures

The primary outcome measure was the sit-to-stand test (STS test). Secondary outcome measures included body weight, body mass index (BMI), and the following blood test parameters: hemoglobin A1c (HbA1c; National Glycohemoglobin Standardization Program), alanine aminotransferase (ALT), aspartate aminotransferase (AST), γ-glutamyl trans peptidase (γ-GT), total protein (TP), blood urea nitrogen (BUN), uric acid (UA), creatinine (Cr), estimated glomerular filtration rate (eGFR), glucose (GLU), total bilirubin (TB), total cholesterol (T-Cho), high-density lipoprotein cholesterol (HDL-Cho), low-density lipoprotein cholesterol (LDL-Cho), non-HDL cholesterol (non-HDL-Cho), triglyceride (TG), and LDL-Cho/HDL-Cho ratio (L/H ratio).

At the conclusion of the final intervention session, participants were asked to complete an open-ended questionnaire to provide feedback on their experience with the HAL-assisted exercise program (Appendices: Figure 4).

Statistical analysis

All measurements were conducted within one week before the intervention started and within one week after the intervention ended. Statistical analyses were performed using JASP 0.18.3 software. Data are presented as mean ± standard deviation (SD). The Wilcoxon signed-rank test was used to compare pre- and post-intervention measurements, with statistical significance set at p<0.05. For the open-ended questionnaire, responses were reviewed, and common themes were identified and summarized to qualitatively describe participant feedback.

## Results

Patient characteristics

Nine patients (4 men, five women) participated in the study. The diagnoses included type 1 diabetes (n=1), type 2 diabetes (n=4), borderline diabetes (n=4), dyslipidemia (n=2), and hypertension (n=2). Six patients had multiple diagnoses, while three had a single diagnosis. All patient characteristics are presented in Table 1.

**Table 1:** Subject characteristics

Patients	Sex	Age	Height (cm)	Weight (kg)	BMI	Diagnosis	Medication
Number 1	Women	62	155	45	18.7	Diabetes (border)	Non
Number 2	Women	38	155	67	28	Diabetes (type Ⅱ),	Non
						Dyslipidemia, others	
Number 3	Women	73	162	60	22.9	Diabetes (type II), Dyslipidemia, others	Tradiance, others
Number 4	Men	72	168	71	25.2	Diabetes (type II), others	Tradiance, others
Number 5	Men	74	176	80.6	26	Diabetes (border), others	Non
Number 6	Women	81	145	61.6	29.3	Diabetes (border)	Non
Number 7	Women	81	156	54.8	22.5	Diabetes (border), others	Non
Number 8	Men	65	172	106.1	35.8	Diabetes (type I), high blood pressure, others	Glubes, others
Number 9	Men	75	169	84	29.4	Diabetes (type Ⅱ), high blood pressure, others	Glubes, others

Implementation status

 All nine participants completed the intervention program without dropping out. The implementation details are shown in Table 2.

**Table 2:** Implementation status

Patients	Weeks of Implementation	Total Number of Sessions	Average Number of Sessions per Week	Average Heart Rate Measurement (1st)	Average Heart Rate Measurement (2nd)	Average Heart Rate Measurement (3rd)	Average Heart Rate Measurement (4th)
Number 1	4	8	2.00	82.5±6.21	85.25±6.58	100±6.78	84.25±4.83
Number 2	4	8	2.00	100.25±6.45	98.5±6.82	102.5±11.05	95.25±5.65
Number 3	5	8	1.60	83.75±1.98	86.25±3.11	89.50±6.82	90.88±6.45
Number 4	5	7	1.40	74.71±7.93	86.29±9.88	96.14±7.58	89.86±7.82
Number 5	8	8	1.00	98.88±8.2	99.38±6.02	100.50±5.58	99.88±5.25
Number 6	4	8	2.00	92.38±2.13	91.13±3.52	92.50±3.12	89.50±3.59
Number 7	4	8	2.00	72.88±4.64	78.38±7.15	82.75±6.50	79.25±6.56
Number 8	7	8	1.14	105.00±5.90	111.88±7.59	113.13±6.36	110.13±5.41
Number 9	5	8	1.60	65.00±3.51	91.88±7.62	108.25±7.92	122.63±8.21

The mean duration of the program was 5.1 weeks, with participants completing an average of 7.9 sessions. The average frequency of sessions was 1.64 times per week. Heart rate monitoring during exercises confirmed that eight out of nine participants maintained their heart rate below 119 beats per minute, indicating appropriate exercise intensity within the target range.

Changes in outcome measures

Significant improvements were observed in several outcome measures following the intervention (Table 3).

**Table 3:** Changes before and after intervention *A paired t-test showed a significant difference before and after the intervention (P<0.05) **A paired t-test showed a significant difference before and after the intervention (P<0.005) ALT: Alanine aminotransferase; AST: Aspartate aminotransferase; γ-GT: Gamma-glutamyl transferase; BUN: blood urea nitrogen; UA: uric acid; eGFR: estimated glomerular filtration rate; T-Cho: total cholesterol; HDL-Cho: high-density lipoprotein cholesterol; LDL-Cho: low-density lipoprotein cholesterol; TG: triglycerides; TP: total protein; TB: total bilirubin; L/H ratio: lymphocytes to heterophils

Blood Test Parameters	Phase	Results
Weight (kg)*	Pre	72.68±15.73
	Post	70.71±15.15
BMI	Pre	27.38±4.02
	Post	26.69±3.94
HbA1c (%)	Pre	6.52±0.73
	Post	6.39±0.65
ALT (mg/dL)	Pre	29.11±33.30
	Post	29.79±19.40
AST (mg/dL)	Pre	25.89±15.54
	Post	26.44±10.24
γ-GT (mg/dL)	Pre	35.33±37.62
	Post	45.44±54.72
TP (g/dL)	Pre	7.17±0.48
	Post	7.56±1.09
BUN (mg/dL)*	Pre	18.62±8.80
	Post	16.75±8.26
UA (mg/dL)	Pre	5.16±1.75
	Post	5.07±1.87
Cr (mg/dL)	Pre	1.28±1.20
	Post	0.92±0.57
eGFR (mL/min)	Pre	66.68±20.58
	Post	65.33±20.71
GLU (mg/dL)*	Pre	121.00±34.26
	Post	101.11±19.23
TB (mg/dL)	Pre	0.76±0.26
	Post	0.69±0.20
T-Cho (mg/dL) *	Pre	200.11±32.85
	Post	182.67±21.45
HDL-Cho (mL/min)	Pre	58.33±11.82
	Post	58.44±14.82
LDL-Cho (IU/L)**	Pre	114.00±28.51
	Post	101.56±24.52
TG (mg/dL)	Pre	198.33±117.02
	Post	145.33±74.50
L/H ratio	Pre	2.04±0.68
	Post	1.90±0.79
Non-HDL-Cho (IU/L)*	Pre	141.78±35.14
	Post	124.22±29.75

**Figure 3:**
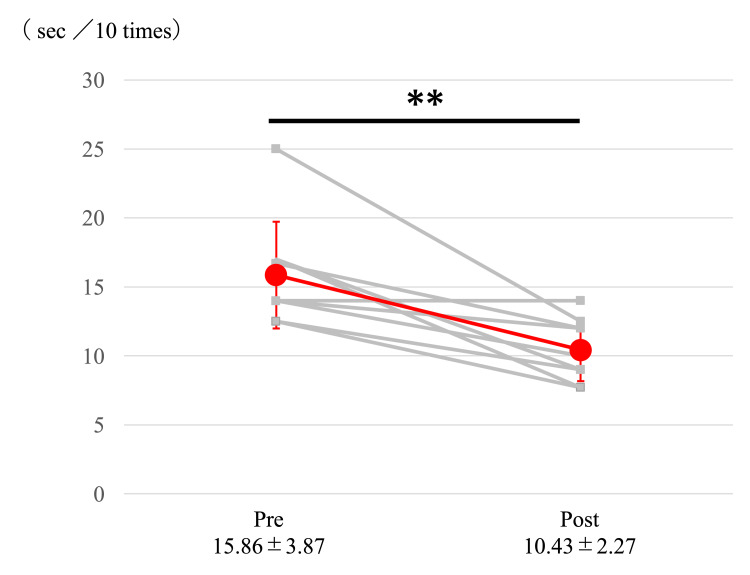
Changes in the STS test **A paired t-test showed a significant difference before and after the intervention (P<0.005)

**Figure 4:**
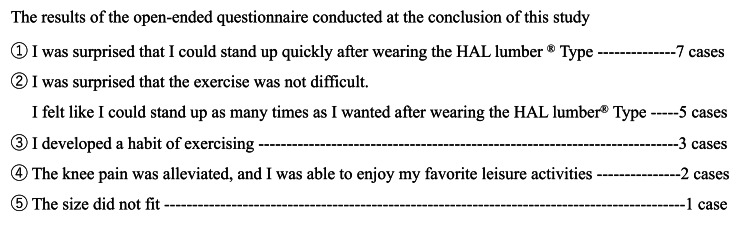
The results of the open-ended questionnaire conducted at the conclusion of this study

Body weight decreased significantly from 72.68±15.73 kg to 70.71±15.15 kg (p<0.05). The STS test showed significant improvement, decreasing from 15.85±3.87 sec/10 times to 10.43±2.27 sec/10 times (p<0.005) (Figure 3). 

Regarding blood parameters, significant improvements were observed in several lipid metabolism markers. Total cholesterol decreased from 200.11±32.85 mg/dL to 182.67±21.45 mg/dL (p<0.05), and LDL cholesterol decreased from 114.00±28.51 mg/dL to 101.56±24.52 mg/dL (p<0.01). Non-HDL cholesterol also showed significant improvement, decreasing from 141.78±35.14 IU/l to 124.22±29.75 IU/l (p<0.05).

Participant feedback

An open-ended questionnaire conducted at the conclusion of the study revealed several key findings. Seven participants reported being surprised by their improved ability to stand quickly while wearing the HAL® Lumbar Type. Five participants noted that the exercise was less strenuous than expected, with many feeling capable of performing multiple repetitions. Three participants reported developing regular exercise habits, and two participants experienced reduced knee pain, enabling them to enjoy leisure activities. One participant reported issues with device fitting.

## Discussion

This pilot study demonstrated that exercise therapy using the HAL® lumbar type could be safely implemented for patients with diabetes. The intervention resulted in significant improvements in both physical function and metabolic parameters, particularly in STS test performance and lipid metabolism markers.

Systematic reviews of exercise therapy for diabetes patients have typically reported significant improvements in HbA1c, blood pressure, and LDL cholesterol following moderate-intensity interventions conducted three times per week for 8-16 weeks [12-16]. However, shorter-term interventions with moderate-intensity exercise, conducted twice weekly for 4 weeks, have also shown metabolic improvements. Notably, Hordern et al. [17] reported changes in resting heart rate, systolic blood pressure, maximal oxygen uptake, BMI, and triglycerides following a 4-week intervention with 150 minutes of moderate-intensity exercise per session. A systematic review by Feng et al. [16] discussed the effects of moderate-intensity HIIT, where high-intensity phases reached 75-95% of maximum heart rate and low-intensity intervals maintained 45-65% of maximum heart rate. Their analysis showed significant effect sizes for glycolipid metabolism (HbA1c, T-Cho, TG, HDL, LDL) with as few as 6-8 sets of moderate-intensity HIIT over two weeks, with an exercise frequency of twice per week or more.

The mechanism underlying improvements in glycolipid metabolism through moderate HIIT remains incompletely understood [18,19]. However, acute responses in skeletal muscle metabolism, including both glucose [17,20,21] and lipid metabolism [22-24], have been well-documented. These acute responses are triggered by various mechanical stimuli to skeletal muscle, leading to rapid energy supply adaptation, reflecting the role of skeletal muscle as an endocrine organ [24-27]. In our study, eight out of nine participants had type 2 or borderline diabetes (non-insulin-dependent diabetes), with a mean HbA1c of 6.52%. This patient profile, combined with our specific intervention protocol, may have particularly favored acute metabolic responses.

Our findings warrant discussion from the following three perspectives.

Patient selection

Our cohort primarily consisted of patients with mild type 2 or borderline diabetes, a population that typically shows strong acute responses to exercise [18,19].

Exercise protocol

Despite using lower intensity than traditional moderate HIIT protocols, our intervention achieved significant metabolic improvements. The HAL lumbar type may have enabled effective exercise performance while maintaining appropriate intensity levels, as evidenced by heart rate monitoring within target ranges.

Errorless motor learning effects

The significant improvement in STS test performance (from 15.85±3.87 to 10.43±2.27 sec/10 times) after only eight sessions suggests potential neural adaptation and motor learning effects. This improvement aligns with previous studies demonstrating that HAL-assisted training can enhance movement patterns and functional performance through optimized errorless motor learning [9-11,28,29]. Participant feedback further supported these findings, with most participants reporting improved movement capability and reduced perceived exertion during exercises. This suggests that the HAL lumbar type may facilitate both physiological adaptation and motor learning, potentially offering a more efficient approach to exercise therapy for diabetes patients.

The strengths of our study include the successful implementation of a novel exercise intervention using the HAL® lumbar type in diabetes patients, with complete adherence from all participants. The significant improvements in physical function (STS test) and metabolic parameters (T-Cho, non-HDL-Cho, LDL-Cho) suggest that HAL-assisted exercise therapy has the potential for observing benefits in physical function and lipid metabolism in a shorter timeframe than might be expected with longer conventional programs. This could have important implications for improving exercise adherence, which is a common challenge in diabetes management.

Several limitations of this study should be acknowledged. First, the small sample size (n=9) limits the generalizability of our findings. Second, the single-arm design without a control group is a significant limitation. It is therefore difficult to definitively attribute the observed improvements solely to the HAL-assisted intervention, as other factors such as the Hawthorne effect, increased attention from study personnel, or concurrent changes in diet or lifestyle cannot be ruled out. Third, outcome assessors were not blinded to the intervention, which could have introduced bias, particularly in performance-based outcomes like the STS test. Fourth, this study did not include a long-term follow-up, so the durability of the observed improvements remains unknown. Fifth, we did not systematically collect data on the duration of diabetes, which prevented subgroup analysis based on disease history. Furthermore, baseline exercise capacity (e.g., VO_2_max) or frailty status was not formally assessed, which would be valuable for characterizing the study population in future research. Finally, other factors such as concurrent exercise habits or clinic visits were not controlled for, which may present an information bias.

## Conclusions

This pilot study demonstrated that exercise therapy using the HAL lumbar type could be safely implemented in patients with diabetes, leading to significant improvements in physical function and lipid metabolism parameters over a relatively short intervention period. The combination of HAL-assisted exercise with conventional diabetes management strategies shows promise as a novel approach to enhance treatment outcomes. Future research with larger sample sizes, control groups, and longer follow-up periods is warranted to confirm these preliminary findings and explore the long-term benefits of this intervention approach.
